# Trained Immunity as an Adaptive Branch of Innate Immunity

**DOI:** 10.3390/ijms221910684

**Published:** 2021-10-01

**Authors:** Vaclav Vetvicka, Petr Sima, Luca Vannucci

**Affiliations:** 1 Department of Pathology, University of Louisville, Louisville, KY 40202, USA; 2 Laboratory of Immunotherapy, Institute of Microbiology, CAS, 142 20 Prague, Czech Republic; sima@biomed.cas.cz (P.S.); vannucci@biomed.cas.czv (L.V.)

**Keywords:** trained immunity, glucan, macrophages, NK cells, basophils

## Abstract

The concept of trained immunity has become one of the most interesting and potentially commercially and clinically relevant ideas of current immunology. Trained immunity is realized by the epigenetic reprogramming of non-immunocompetent cells, primarily monocytes/macrophages and natural killer (NK) cells, and is less specific than adaptive immunity; therefore, it may cross-protect against other infectious agents. It remains possible, however, that some of the observed changes are simply caused by increased levels of immune reactions resulting from supplementation with immunomodulators, such as glucan. In addition, the question of whether we can talk about trained immunity in cells with a life span of only few days is still unresolved.

## 1. Types of Immunity of Multicellular Organisms

Contrary to plants and invertebrates, the defense of body integrity and its internal environment of all vertebrates can be divided into constitutive (natural, or nonspecific) and acquired (adaptive, or specific) immunity. The nonspecific immunity is innate, which means that it manifests itself fully from birth and acts for the entire life of an individual. Specific immunity, on the other hand, takes more time to develop until it becomes fully functional.

Adaptive immunity gradually creates a more precisely targeted response to the “nonself” foreign antigenic structures that the individual encounters during its life. In addition, it stores mechanisms of this specific response in the form of immunological memory, which can last for the entire life.

Constitutive immunity recognizes foreign structures by the germline-encoded molecular sensors, pattern recognition receptors (PRRs) [1,2], that evolve before the tissues, cells, and effector mechanisms of adaptive immunity. PRRs are expressed mainly by dendritic cells, macrophages, monocytes, neutrophils, and epithelial cells [3]. They bind to molecular structures expressed on the surfaces of pathogenic organisms, which potentially endanger the integrity of an individual’s uniqueness, called pathogen-associated molecular patterns (PAMPs) (for review, see Santoni et al., 2015 [4]) and danger-associated molecular patterns (DAMPs) [5,6]. The binding reaction of PRRs with PAMPs and DAMPs is very effectively realized by engulfing invading pathogenic microorganisms by phagocytic cells, followed by killing them via production of oxygen species, numerous enzymes, and cytokines. Both ways end with the destruction of the threatening agent.

For decades, immunologists have assumed that constitutive immunity is not endowed by any type of immunological memory. In recent years, however, conclusive evidence has accumulated demonstrating that innate immunity may also possess some adaptation ability. It recognizes and remembers the non-self, the foreign molecular patterns not only in vertebrates but also in invertebrates [7,8,9,10], and even in plants, and is termed “systemic acquired resistance” [11,12,13] (for review, see Gourbal et al., 2018 [14]).

In invertebrates, which represent up to 97% of the total biodiversity on Earth [15], adaptive immunity is lacking, and protection of their internal milieu depends solely on their innate immune systems by which they recognize PAMP and DAMP pathogenic structures and mount a defense against them. However, there is supposition that invertebrates must also be endowed by some type of immunological memory because, without the ability to remember their previous meetings with threatening pathogens and their more rapid disposal, their survival and evolutionary success could hardly be imagined. It was recently demonstrated that the first infection in invertebrates induces a more rapid and stronger defensive response against the secondary infection by the same pathogen [16,17]. Moreover, it was also observed that certain infections and vaccinations can induce specific protection mediated by innate immunity mechanisms (also against other pathogens) [18,19]. This type of defense has been observed in all animals endowed by an innate immunity, including vertebrates, in which an evolutionary new form of defense, specific adaptive immunity, developed [9].

**Figure 1:**
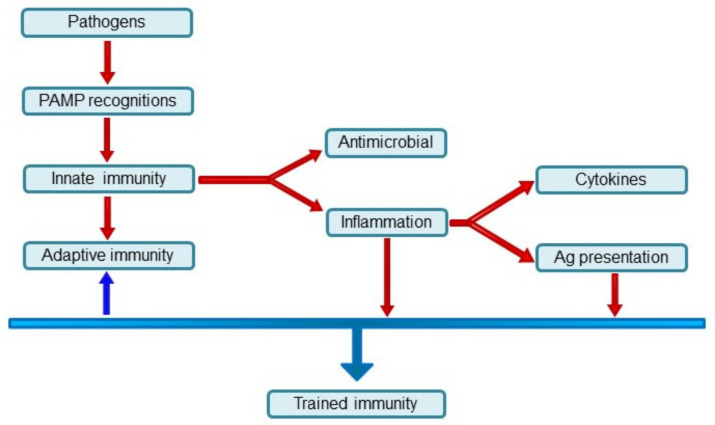
An overview of immune response. PAMP recognition initiates both parts of the immune response. Antimicrobial response followed by an inflammatory response plays a role in the development of trained immunity.

These observations have led to the hypothesis that innate immunity could be influenced by previous encounters with PAMPs and DAMPs or other products of pathogenic microorganisms and develop mechanisms to remember these structures (Figure 1). This ability of innate immunity to display, by some degree, innate immune memory was coined “trained immunity” by M. G. Netea in 2011 [20]. Trained immunity is sometimes also called “innate immune memory” and is not specific.

The germline-encoded PRRs of innate immune systems are activated by numerous environmental factors, including PAMPs and DAMPs of microbial and viral origin. A vast majority of those factors are food-borne antigenic substances. PRRs trigger activation of inflammasomes formation, which are needed for the elimination of harmful stimuli and for the recovery of damaged tissues. The inflammasomes contain multimeric protein complexes that activate inflammatory response and are defined by their PRRs. Five types of PRRs forming inflammasomes have been described: the nucleotide-binding oligomerization domain (NOD), leucine-rich repeat (LRR)-containing proteins, (NLR) family members NLRP1, NLRP3, and NLRC4, as well as absent-in-melanoma 2 (AIM2) and pyrin [21,22].

The NLRP3 type of inflammasome is important for immune response against bacterial, fungal, and viral infections. In addition, it is interconnected with some chronic, non-communicable diseases, such as atherosclerosis, Alzheimer disease, diabetes 2, gout, auto-inflammatory diseases, and atherosclerosis [23,24]. In vitro cellular experiments revealed that innate immune response may be induced by oxidized low-density lipoprotein (oxLDL) cholesterol particles, which are known to trigger innate memory immune response in human monocytes [25]. This means that the composition of some unhealthy diets, among those especially so-called Western diets, could trigger long-lasting inflammation, which ultimately results in the induction of innate immune memory and trained immunity [26].

Conversely, when a vertebrate encounters a potential threat to their internal integrity disruption through the activation of clones of lymphoid cells, it recognizes specific antigens from the invading pathogens and consequently rearranges its gene segments responsible for the production of immunoglobulins (antibodies), binding those antigens. From an evolutionary point of view, the adaptive immune system, in which immunocompetent cells of lymphoid origin mediate immunological memory, is a relatively recent development in the predecessors of vertebrates from 500 million years ago [27,28,29].

## 2. Cells Mediating the Trained Immunity

Trained immunity differs from classical immunological memory of adaptive immunity in several important respects. It is performed by a row of cellular populations differing from each other by their origin and effector functions. They are primarily myeloid cells, monocytes and macrophages, NK cells, and dendritic cells. Even innate lymphoid cells (ILCs) are functionally different from those involved in classical immunological memory (for review, see Netea, et al. 2020 [30]).

### 2.1. Basophils

Basophils are capable of capturing antigen specific IgE antibodies via expression of surface IgE receptors, resulting in fast capture and clearance of the pathogen [31]. The reason for this increased response is the ability of specific antigen recognition by IgE antibodies, i.e., antibodies produced by adaptive immune mechanisms [32]. In hookworms, basophils have been reported to protect against reinfection with *Nippostrongylus brasiliensis,* independently of mast cells and memory T helper 2 (Th2) cells [33]. There is a low probability of basophils acquiring a “memory” phenotype after their first encounter with a parasite, because they have a life span of several dozen hours and hence, could not persist until a secondary exposure [34]. Yet the presence of a parasite could induce changes in hematopoiesis [35], leading to epigenetic and transcriptomic changes in the progenitor cell subpopulation of basophils, which induces a long-term protective innate immune memory, a common feature of trained immunity [36,37]. However, these findings all need to be tested in future experiments to definitively prove that these cells possess the capability to develop trained immunity.

### 2.2. Neutrophils

Neutrophils also represent a short-lived subpopulation of myeloid cells. The main cell type is granulocytes, which are primarily engaged in bacterial infection, during which they act as phagocytes and kill the pathogens, produce reactive oxygen species, and release neutrophil extracellular traps and different types of proteases [38]. They form the innate immune compartment which, after helminth infection, acquires an alternative transcriptional profile, allowing them to induce long-term immunity with many features resembling the trained immunity in lung-resident macrophages, which protect during secondary infection [39]. This innate memory of activated macrophages is associated with the subpopulation or innate lymphoid cells of group 2 and CD4 T cells in the lung [40]. It could be assumed that the mechanism of induction of trained neutrophils is very similar to that in basophils: as invading pathogens induce neutrophil progenitors, the epigenetic changes cause the development of an innate type of immune memory. As with basophils, these conclusions need to be supported by further study.

### 2.3. Mononuclear Phagocytes

Besides the main effector function of macrophages and phagocytosis, they also produce reactive oxygen species and soluble cytokines and alert other cells to the presence of an infectious agent [41]. Macrophages exert features of immune memory during secondary immune response against a bacterial infection and adoptive transfer of macrophages from immunized donors to naïve recipients, sufficiently conferring protection. Furthermore, the inhibition of lymphocyte functions by cytostatics does not impair the induced protection [42] (more details below). In one of the few studies focused on the duration of trained immunity, stimulation by β-glucan was found to be rather short-lived, and no effects were found after 20 days [43].

### 2.4. Innate Lymphoid Cells

The recently discovered lymphoid cells of innate immunity form a subset of a large family of lymphoid cells. They contribute to overall immune reactions by secreting regulatory factors, for example, the cytokines, which affect the other cell types. ILCs are primarily resident and abundant in lymphoid and non-lymphoid tissues, such as at the mucosal barriers of respiratory and gastrointestinal tracks, where they are exposed to the immunogens, namely foreign antigens, PAMPs and DAMPs of pathogenic microorganisms, viruses, nutritional components, and substances evoking allergic reactions [44]. They are also minimally present in the peripheral blood [45]. NK cells and lymphoid tissue inducer cells are considered to be ILCs because they express common features and activities and produce interferons [46]. ILCs share common progenitors with other lymphoid cell populations but they do not express the RAG-dependent antigen receptors (BCR, TCR), even if they can produce an array of cytokines similar to other T helper cell subsets. ILCs function in lymphoid organogenesis, tissue remodeling, antimicrobial immunity, and inflammation and are critical in the first line of immune defense. They are classified into three major subclasses: ILC1s, ILC2s, and ILC3s. The ILCs are involved in lymphoid tissue formation, mucosal immunity, and inflammation and are important for immunity against helminth parasites. The main cell subpopulation among ILCs are the NK cells which, upon activation, not only exert cytotoxic activity but also produce interferon gamma (IFNγ) [47]. ILCs were thought to lack immune memory, but growing evidence suggests otherwise [48].

### 2.5. NK Cells

Primed NK cells differentiate into some form of “memory cells”, infiltrating various tissues, and after a secondary meeting with the same antigens, produce protective cytokines. It has been reported that NK cells display a type of memory and, secondarily, prime bone marrow myeloid cells, representing the effectors of trained immunity [49,50,51,52].

In comparison to the cells of adaptive immunity, in which their antigenic specificity lies in the rearrangement of relevant genes and consequent predetermined differentiation and proliferation of lymphoid cell lines, the increased responsiveness of effector cells of trained immunity is not antigen-specific. This is realized by means of epigenetic reprogramming signals affecting transcription factors, which control cell functions, especially metabolism, and further production of effector molecules. Induced trained memory of functionally altered cells of innate immunity may be long lasting, i.e., it may persist for a long time after the initial stimulus and when the pathogen, from which the stimulus evoked, is no longer present.

Genome-wide epigenetic changes resulting in the elevation of the antimicrobial functions on involved cells are detectable for a prolonged time and, in some cases, up to one year [53]. However, most of the abovementioned cellular populations are short lived with the average half time of several days [37,54], so it is not known what mechanism is responsible for maintaining the immune memory of trained immunity over long periods. This type of memory may be stored within bone marrow progenitor cells as indicated by vaccination studies with Bacillus Calmette–Guérin (BCG) and especially with β-glucan.

Metabolomic and shotgun lipidomic evaluation of bone marrow progenitor cells from β-glucan-stimulated mice reported lower levels of metabolites involved in arachidonic and linoleic acid metabolism [37]. Similarly, glucan stimulated significant alterations in other metabolic pathways, mainly in cholesterol mechanism and glycolysis. Some studies found an elevation of key enzymes in the tricarboxylic acid cycle and glycolytic pathway [55]. Based on the findings that H3 histone lysin 4 monomethylation is important for trained immunity, the role of Set7 methyltransferase was evaluated in glucan-induced trained immunity. The study found Set7-dependent changes in gene expression upon glucan treatment, suggesting that Set7 is a key regulator of trained immunity, at least in cases of glucan-mediated trained immunity [56]. This could be a result of Set7 lysine methyltransferase regulating plasticity in oxidative phosphorylation. There is an excellent review by Quintin 2019 [57] for readers seeking a detailed summary of specific genes involved in the induction of trained immunity.

The possibility that these metabolic changes are involved in the reaction to diverse stimuli is often discussed [58,59] but, so far, these discussions are more speculative than confirmed. Despite numerous observations of metabolic changes in trained immunity, there is no clear explanation of how described alterations in glucose or fatty acid metabolism improves immune reactions.

## 3. Induction of Trained Immunity

Originally, evidence for the existence of trained immunity in vertebrates was obtained from experiments with mice, which were protected against lethal bacterial infection with *Staphylococcus aureus* by nonspecific substances, such as β-glucan [60,61]. Other substances, such as muramyl dipeptide peptidoglycan components and oligodeoxynucleotides containing CpG motifs and flagellins, are protected against infection with *Toxoplasma gondii* and *Escherichia coli* meningitis. The basic idea behind these findings was that certain challenges promoted heightened response of myeloid cells upon subsequent infection with the same (and, in some cases, different) pathogens. However, many authors consider the experiments with *Candida albicans* as the first description of trained immunity [62].

Furthermore, flagellin can induce protection against *Streptococcus pneumoniae* and rotavirus [63,64,65,66]. In addition, some proinflammatory cytokines may induce trained immunity [67]. In experimental studies in which the mice were immunized with the BCG vaccine, the protection against secondary infection with *C. albicans* and *Schistosoma mansoni* was observed [68,69]. When athymic and recombination-activation-gene (*RAG1*)-deficient mice (that cannot rearrange their antigen receptors) were infected by a lowly virulent *C. albicans*, the same type of protection was reached. This provides further evidence that trained immunity was not dependent on the mechanisms of adaptive immunity [42,62], but the animals were protected against reinfection by macrophage activation and cytokine production [70,71]. The protective effects, which are not mediated by mechanisms of adaptive immunity but are realized mainly by macrophages, may also be induced by various pathogenic organisms, such as herpes virus-induced resistance against *Yersinia* and *Listeria* [72], bacteria [71], and the helminth parasite, *Nippostrongylus brasiliensis* [39].

BCG vaccination in humans equally activated trained immunity mechanisms, such as higher activity of monocyte–macrophage cell lineage, which led to higher protection against some infections such as yellow fever [73] and malaria [74]. In both adults [53,75] and infants [76,77], these effects lasted for several months. BCG vaccination also is reported to have induced an antitumor type of trained immunity, mainly in cells of the monocyte–macrophage lineage, which could be effective during therapy of some malignancies, including bladder cancer [78], melanoma [79], leukemia [80], and lymphoma [81].

Most studies have focused on changes in cells upon primary activation via numerous modulators, such as glucan, BCG, or monosodium urate crystals. Few tried to determine if clinical infection induced trained immunity in humans. Using a *Plasmodium falciparum* infection model, monocyte response showed biphasic pattern—low levels of inflammatory cytokines followed by a strong increase of interleukin 6 (IL-6) and tumor necrosis factor alpha (TNF-α) secretion 36 days after the original stimulus [82]. Epigenomic and transcriptomic changes were observed at both timepoints.

To date, trained immunity has been studied mostly in rodents and humans. Teleost fish are the first vertebrates with a fully developed defense system, so the possible presence of trained immunity cannot be overlooked. Using a carp model of head kidney-derived macrophages, clear evidence of metabolic reprogramming and higher phagocytosis and cytokine and reactive oxygen and nitrogen species release have been observed [83]. An interesting hypothesis was raised by Quintin [57], who suggested that there are two immunologically opposite parts of the innate immunity memory—tolerance and trained immunity—and these parts might be epigenetically or mechanistically mirrored.

Although most experimental models of trained immunity have utilized pathogens, it is plausible that life-long exposure to inflammation also affects trained immunity. It is possible that cumulative exposure of an individual provides the main establishment of immune training and/or immunotolerance. The final results might be based on the balance between the duration of the dose and an order of exposure. In addition, trained immunity might be involved in altering inflammatory disease development [84]. Major mechanisms affecting systemic chronic inflammation (such as diet, infections, and pollution) can be found also in induction of trained immunity, suggesting possible connections [85].

## 4. β-Glucan

**Figure 2:**
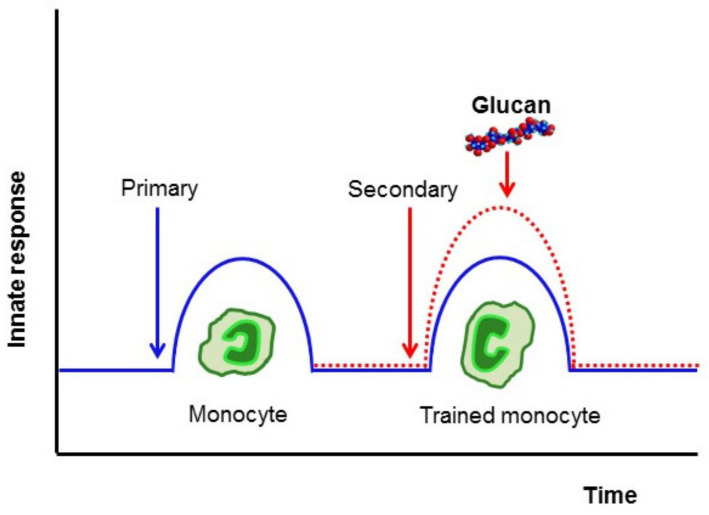
Basic concept of trained immunity. Dotted line shows classical concept, full line shows trained immunity.

β-Glucans represent biological response modifiers. They exert a variety of biological and immunopharmacological properties [86,87,88]. It should be taken into account that glucans are structurally variable molecules and may contain an array of impure compounds. It is not known to what extent this structural variation and the quantity of contaminating substances modify the β-glucan immune effects. However, it is certain that some structures of β-glucan act as a PAMPs and, similarly to BCG, enhance innate immunity (Figure 2), particularly the trained immunity by induction of epigenetic reprogramming bone marrow hematopoietic cells, and their myelopoietic differentiation into effector cell populations, mainly monocytes and macrophages [36,89,90] (for review, see Sima et al., 2020 [91]). In addition, in vitro experiments have shown that the stimulation of monocytes by either BCG or β-glucan resulted in elevated levels of the same cytokines, mainly IL-6, interleukin 10 (IL-10), and TNF [92]. This capacity to change the cytokine production was found to be identical in cells isolated from neonates or adults. Detailed analysis of monocyte–macrophage differentiation upon β-glucan addition found a new population of long-lived monocyte-derived macrophages, but no clear differences in their function, only some elevated activities [89].

The expansion of cells starts with progenitors and is subsequently followed by elevated signaling by innate modulators, such as granulocyte–macrophage colony-stimulating factor (GM-CSF) and interleukin 1 beta (IL-1β), or changes in cholesterol synthesis and glucose metabolism. Based on their role in trained immunity, β-glucans are sometimes called “prototypical trained immunity–inducing agonists” [37]. A detailed study revealed that β-glucan can induce significant protective trained immunity against *Mycobacterium tuberculosis* by histone modification at a gene promoter level. This subsequently resulted in an elevated secretion of IL-1. This finding was confirmed in IL-1R–lacking mice [93]. Molecular analysis of the β-glucan role in the induction of trained immunity showed that β-glucan used as a primary stimulus (and lipopolysaccharide (LPS) as secondary) induced a gene expression signature involving a PI3K/AKT signaling pathway, resulting in the elevated secretion of GM-CSF, upregulation of 4.5 LIM-only protein 2 and upregulation of Dectin-1 expression [94]. Most of these studies used yeast-derived β-glucan for the induction of trained immunity. However, the use of oat-derived β-glucans offered similar results, with changes in mRNA expression and secretion of IL-6 and TNF-α [55].

One of the most interesting studies focused on a theory that β-glucan-induced trained immunity can start antitumor activity [95]. Prophylactic treatment with glucan caused lower tumor growth (which has been observed repeatedly in other studies), but adaptive transfer of trained neutrophils into naïve animals suppressed cancer growth again. Detailed evaluation found that transcriptomic and epigenetic rewiring of neutrophils and entire granulopoiesis toward an anticancer phenotype [83]. If confirmed, these findings might open a new window into cancer treatment, as β-glucan is already being used as a supplement or anticancer drug [96]. Kalafati’s research might result in recommending use of glucan as a prophylactic. Numerous studies have confirmed the anticancer effects of glucans as an immune stimulant (for review see Wu et al., 2021 [97]), but as a confirmed inducer of trained immunity, β-glucan supplementation has gained another meaning [98].

Some studies have offered results which are difficult to interpret. In vitro experiments found a significant role of Toll-like receptor 10 in the modulation of β-glucan-induced trained immunity but also reported no role for this receptor in in vivo induction of trained immunity [99]. These findings might result from differences between direct interaction of glucan on monocytes in vitro and situation in situ, or from differences between in vitro and in vivo doses of β-glucan. Similarly, the findings of β-glucan efficacy against leptospirosis with improved survival, enhanced expressions of TLRs, and secretion of IL-1 and iNOS [100] seem to be more in agreement with the previously published effects of β-glucan on a parasite [101] than with proof of trained immunity. Using a model of *Leishmania braziliensis* infection, glucan-induced trained immunity protected the animals by augmented release of IL-32 [102]. One of the rare human studies found long-term functional changes in malaria infections, resulting in an increased IL-6 and TNF-α response [82].

**Figure 3:**
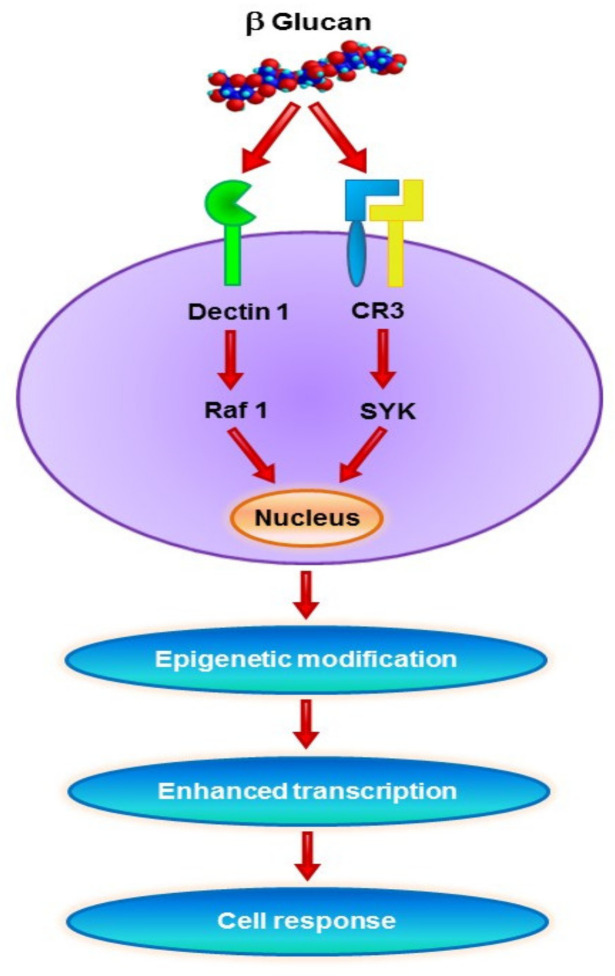
Potential cell signal transduction pathways involved in the generation of trained immunity. Functional reprogramming is associated with epigenetic changes.

β-Glucan-induced trained immunity has been studied mostly in mice (Figure 3). However, experiments using additional species found that its action is probably more general. In dogs, β-glucan improved the action of an inactivated rabies vaccine by stimulation of both B and T lymphocytes [103]. β-Glucan was also found to induce significant trained immunity in chicken monocytes, similarly to mammals [104]. So far, no epigenetic changes have been found, but this is probably due to the limited number of studies using avian models. A more detailed study found some training of innate immunity in chicken, particularly in increased mRNA levels of IL-1 and hypoxia-inducible factor alpha (HIF-1α), but there were significant differences between monocytes isolated from layers and broilers. In both cases, the effects on disease resistance were not tested, making these more direct effects of β-glucan on immune reactions than real trained immunity [105]. Similar results were also found in a study of β-glucan-induced trained immunity in newborn goats [106].

The findings of trained immunity in teleosts were mentioned above. A detailed study using turbot (*Scophthalmus maximus*) found that β-glucan induced significant metabolic changes, including in glucose, adenosine triphosphate (ATP), and lactate levels, as well as fatty acid and glucose metabolism, leading to lowering of mortality in subsequent infection [107]. Other studies, however, did not describe any epigenetic changes [108]. Contradictory results might be caused by several different receptors needing to be involved, especially Dectin-1 and TLR2/6. Not all glucans bind to all these receptors. In teleost, more than 21 different Toll-like receptors have been identified [109], but only little is known about their binding to β-glucan.

## 5. Conclusions

As more studies have confirmed the existence of trained immunity, the classical binary classification of immune memory has become obsolete. Summarized together, trained immunity effects induced by microbial products (such as BCG, β-glucans, and lipopolysaccharides) are accompanied by a more effective cytokine response, which could lead to improved antiviral protection, even from the coronavirus disease, COVID-19 [110] (for a review, see Netea et al. 2020 [111]). β-Glucan-induced trained immunity has been suggested as an effective way to boost immune response against COVID-19 infection and even to abrogate symptoms [112].

Trained innate immunity represents an evolutionary conserved phenomenon among some plants, some invertebrates, and all tested vertebrate species. It is induced after a primary meeting with a pathogen and confers protection against a secondary infection independently on the mechanisms of adaptive immunity. Trained immunity is realized by epigenetic reprogramming of non-immunocompetent cells, primarily macrophages and NK cells, and is less specific than adaptive immunity, therefore offering cross-protection. We cannot overlook, however, the possibility that some of the observed changes are simply caused by elevated levels of immune reactions caused by supplementation with immunomodulators. In addition, the question of whether we can talk about trained immunity in cells with a life span of only a few days is still unsolved.

As some experimental designs are open to this interpretation, deeper and more detailed studies on the relationship between epigenetic and metabolic changes and changes in the levels of immune reactions are necessary. In addition, some studies have suggested the role of Dectin-1 [113], but the involvement of complement receptor type 3 (CR3) and how the glucan effects are manifested in cells lacking Dectin-1 remain unknown.

The various actions of trained innate immunity on precursor cells have a strong potential for therapeutic use, particularly in infected and myelosuppressed individuals. In addition, the improvements of effects of some vaccines offer other potential use of β-glucan as an inductor of trained immunity, suggesting novel uses of a traditional therapeutic.

## Data Availability

Not applicable.
